# The Biochemical Composition and Quality of Adult Chinese Mitten Crab *Eriocheir sinensis* Reared in Carbonate-Alkalinity Water

**DOI:** 10.3390/foods13030362

**Published:** 2024-01-23

**Authors:** Shihui Wang, Liang Luo, Rui Zhang, Kun Guo, Zhigang Zhao

**Affiliations:** 1 Key Open Laboratory of Cold Water Fish Germplasm Resources and Breeding of Heilongjiang Province, Heilongjiang River Fisheries Research Institute, Chinese Academy of Fishery Sciences, Harbin 150070, China; hrfriwsh@yeah.net (S.W.); luoliang@hrfri.ac.cn (L.L.); zhangrui@hrfri.ac.cn (R.Z.); guokun@hrfri.ac.cn (K.G.); 2 Engineering Technology Research Center of Saline-alkaline Water Fisheries (Harbin), Chinese Academy of Fishery Sciences, Harbin 150070, China

**Keywords:** *Eriocheir sinensis*, saline–alkaline, aquaculture, edible yield, mineral element

## Abstract

Saline–alkaline aquaculture has become an important breakthrough in expanding the space available for aquaculture in China. However, the biochemical composition and quality of *Eriocheir sinensis* reared in carbonate alkalinity water are still unclear. Therefore, this study investigated the edible yield, coloration, and nutritional and flavor quality of *Eriocheir sinensis*. A significantly lower gonadosomatic index (GSI), meat yield (MY), and total edible yield (TEY) were detected in intensive pond (IP) samples than those in semi-intensive reed wetland (SIWR) (*p* < 0.05). Six color parameters in the hepatopancreas (*p* < 0.05) differed between IP and SIRW. The contents of crude protein and fat in the female hepatopancreas of IP were significantly higher than those in SIRW (*p* < 0.05). The concentrations of total monounsaturated fatty acids (∑MUFA), total essential fatty acids (∑EFA), and hypocholesterolaemic/hypercholesterolaemic ratio (h/H) in the female edible tissues checked were higher in IP than those in SIRW, with significant differences including ∑MUFA in the hepatopancreas and ovary, ∑EFA in the muscle, and h/H in the ovary (*p* < 0.05). Higher total free amino acid (∑FAA) contents of muscle were detected in SIRW than that in IP samples. Significantly higher K, Ca, Mg, Fe, and Zn contents in the ovary were detected in SIRW samples compared to IP (*p* < 0.05). Overall, *Eriocheir sinensis* reared in carbonate-alkalinity water is an important source of nutrients.

## 1. Introduction

Saline–alkaline land, accounting for 0.95 billion ha, covers approximately 7.26% of the total land area worldwide [1], of which 99.13 million ha saline–alkaline land are located in China, including approximately 45.87 million ha of low-lying saline–alkaline water mainly distributed in northeast, northwest, and coastal areas [2]. Salinity and carbonate alkalinity, serving as the most significant stressors in saline–alkaline water, have a substantial impact on the growth, survival, reproduction, and quality of aquatic animals [3,4,5]. The physiological metabolism, osmoregulation, and intestinal microbiota of aquatic animals can be significantly influenced by high saline–alkaline concentrations [6,7,8,9,10,11]. Therefore, not all aquatic animals can be reared in the saline–alkaline water. Only a few species, such as Nile tilapia *Oreochromis niloticus* [9], crucian carp *Carassius auratus* [10], Chinese mitten crab *Eriocheir sinensis* [5], naked carp *Gymnocypris przewalskii* [11], Bulatmai barbel *Luciobarbus capito* [12], and White shrimp *Litopenaeus vannamei* [13] are domesticated for saline–alkaline water culture. Due to shrinking aquaculture space, expanding new aquaculture space for aquaculture development is thus essential, especially in saline–alkaline water.

The saline–alkaline water distributed in China mainly comprises three types, including chloride, carbonate, and sulfate, where the main ions are Na^+^, K^+^, Ca^2+^, Mg^2+^, Cl^−^, CO_3_^2−^, HCO_3_^−^, OH^−^ and SO_4_^2−^, separately [14]. Meanwhile, different types of saline–alkaline water also have different ionic compositions. Cl^−^ is the main anion of chloride alkalinity, while CO_3_^2−^ and HCO_3_^−^ are in carbonate alkalinity, and SO_4_^2−^ is observed in sulfate alkalinity [15]. Among these, Daqing area in Heilongjiang province is a typical saline–alkaline wetland with abundant carbonate alkalinity (NaHCO_3_) located in the western Songnen plain of China, where high carbonate alkalinity and pH in the saline–alkaline water are characteristics of the environment. High carbonate alkalinity can reduce the concentration of H^+^ in saline–alkaline water due to the high pH, thereby leading ammonia (NH_3_) toward the direction of an equilibrium state of NH_4_^+^ + OH^−^ ⇌ NH_3_·H_2_O and causing NH_3_ poisoning in aquatic animals [16,17,18]. Consequently, the growth and quality of aquatic animals may be influenced significantly.

The Chinese mitten crab, *Eriocheir sinensis*, is an important aquatic animal with high economic and nutritional values. In addition, its aquaculture yield reached 815,318 t in 2022 [19]. *E. sinensis* is a migratory aquatic animal that grows in freshwater until it reaches sexual maturity and reproduction occurs in brackish water, thereby leading to high salinity tolerance [20]. Recent studies have also illustrated that *E. sinensis* has high carbonate-alkalinity tolerance characteristics [5]. However, the edible yield and quality of *E. sinensis* reared in carbonate-alkalinity water are still unclear. Therefore, the aim of this study was to investigate the edible yield, coloration, and nutritional and flavor quality of *E. sinensis* reared in carbonate-alkalinity water.

## 2. Materials and Methods

### 2.1. Experimental Set Up and Culture Management

The *E. sinensis* was reared in carbonate-alkalinity water from a local aquaculture demonstration farm (124.62° E, 45.70° N) in Zhaoyuan City of Heilongjiang Province, China. The culture experiment began on 1st May 2021 and was completed on 30th September 2021. The megalopa Guanghe No. 1 originated from Panjin Guanghe Crab Industry Co., Ltd., Panjin, China, with an juvenile average body weight of 5.32 ± 0.26 g/ind. These juveniles were reared in carbonate-alkalinity water at a stocking density of 15,000/ha, and the water quality parameters were as follows: intensive pond (IP), salinity 0.68 ± 0.05 ppt, carbonate alkalinity 8.48 ± 0.32 mmol/L, pH 8.72 ± 0.04; semi-intensive reed wetland (SIWR), salinity 0.56 ± 0.02 ppt, carbonate alkalinity 8.88 ± 0.04 mmol/L, and pH 8.65 ± 0.04. The IP was transplanted with Canadian pondweed, *Elodea canadensis*, while the SIWR was planted with natural *Phragmites australis*. During the culture stage, the juveniles were fed twice a day at 8:00 a.m. and 5:00 p.m. with a commercial formulated diet (crude protein ≥ 39.0%, crude fat ≥ 5.0%, moisture ≤ 12.0%, ash ≤ 18.0%. Nantong Charoen Pokphand Co., Ltd., Nantong, China). The feeding amount accounted for approximately 2% of the total body weights.

### 2.2. Sample Collection and Dissection

All *E. sinensis* procedures in this study were conducted according to the Guidelines for the Care and Use of Laboratory Animals of Heilongjiang River Fisheries Research Institute (HRFRI), Chinese Academy of Fishery Sciences (CAFS), Harbin, China. The *E. sinensis* used in the present study were reviewed and approved by the Committee for the Welfare and Ethics of Laboratory Animals of HRFRI, CAFS (Approval code: 20210910-002; approval date: 10 September 2021). On 4 and 29 September 2021, a total of one hundred mature *E. sinensis* (♀:♂ = 1:1) reared in IP and SIWR were collected, respectively. Subsequently, these alive *E. sinensis* were transported to HRFRI, CAFS, accurately weighed with an electronic balance (JA2002, Shanghai Puchun measuring instrument Co., Ltd., Shanghai, China), and then the carapace length and carapace width were also measured with a Vernier caliper (605, Harbin measuring tools and cutting tools Co., Ltd., Harbin, China). The anatomical procedures were followed according to the previous study [5]. The hepatosomatic index (HSI, %), gonadosomatic index (GSI, %), meat yield (MY, %), total edible yield (TEY, %), and condition factor (CF, g/cm^3^) were also referenced in the Wang et al.’s study [5] and calculated according to the following Equations (1)–(5):

GSI (%) = 100 × Gonad weight/Body weight(1)

HSI (%) = 100 × Hepatopancreas weight/Body weight(2)

MY (%) = 100 × Muscle weight/Body weight(3)

TEY (%) = GSI (%) + HSI (%) + MY (%)(4)

CF (g/cm^3^) = Body weight/Carapace length^3^ (5)

### 2.3. Measurements of Color and Nutritional Parameters

The color parameters L* (brightness), a* (redness), and b* (yellowness) of carapace, hepatopancreas, and female gonad (ovary) between IP and SIRW samples were measured by a colorimeter (CR-400, Konica Minolta, Marunouchi, Tokyo, Japan). The measured method was carried out according to Long et al. ’s study [21]. The overall collected samples of each edible tissue were randomly selected and combined into three duplicate samples. The proximate composition, fatty acids, free amino acids, and mineral elements of *E. sinensis* reared in IP and SIRW were measured as reported in a previous study [22]. The moisture was checked by using a vacuum freeze-dryer (FD-1A-50, Biocoll, Beijing, China) at −50 °C vacuum freezing to a constant weight. The crude protein (Kjeldahl method) and ash at 550 °C were extracted by muffle furnace burning by using the AOAC method [23]. The crude fat was extracted by using the Soxhlet extraction method. An Agilent 7890B-5977A gas chromatograph (GC–MS, Agilent Technologies Co., Ltd., Santa Clara, CA, USA) was used for the determinations of fatty acids. An automatic amino acid instrument (L-8800, Hitachi Co., Ltd., Tokyo, Japan) was used for the free amino acid analysis. The taste activity value (TAV) was evaluated as the ratio between the determined FAA content and its threshold [24]. The mineral elements were performed by using an inductively coupled plasma-mass spectrometer (7500, ICP–MS, Agilent Technologies Co., Ltd., PA, USA). The hypocholesterolaemic/hypercholesterolaemic ratio (h/H), index of atherogenicity (AI), and index of thrombogenicity (TI) [25] were calculated using the following Equations (6)–(8):

h/H = ∑(18:1n9, 18:1n7, 18:2n6, 18:3n6, 18:3n3, 20:3n6, 20:4n6, 20:5n3, 22:4n6, 22:5n3, 22:6n3)/∑(14:0,16:0)(6)

AI = (12:0 + 4 × 14:0 + 16:0)/(∑n−6 PUFA + ∑n−3 PUFA + ∑MUFA)(7)

TI = (14:0 + 16:0 + 18:0)/(0.5 × ∑MUFA + 0.5 × ∑n−6PUFA + 3.0 × ∑n−3PUFA + n−3/n−6 PUFA)(8)

### 2.4. Statistical Analysis

The results are presented as the mean values ± standard error (SE). SPSS 22.0 software (SPSS Inc., Chicago, IL, USA) was used for statistical analysis. Independent samples *t*-test was used to determine the differences between IP and SIWR. In comparison tests, *p* < 0.05 was regarded as statistically significance, and *p* < 0.01 was regarded as extremely statistically significance.

## 3. Results

### 3.1. Total Edible Yield

**Figure 1:**
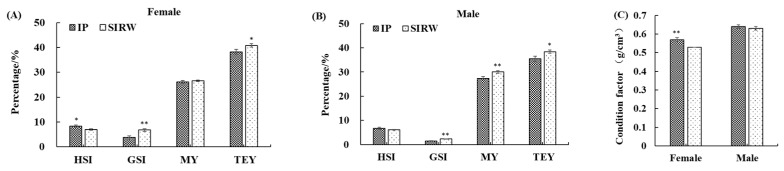
The edible yield (% body weight, (**A**,**B**)) and condition factor (%, (**C**)) of adult *Eriocheir sinensis* reared in carbonate-alkalinity water. Data are presented as means ± standard error (SE) (n = 25). * denotes significant difference (*p* < 0.05), ** denotes extreme significant difference (*p* < 0.01). IP, *Eriocheir sinensis* reared in intensive pond; SIRW, *Eriocheir sinensis* reared in semi-intensive reed wetland; HSI, hepatosomatic index; GSI, gonadosomatic index; MY, muscle yield; TEY, total edible yield.

The edible yield and condition factor of adult *E. sinensis* reared in carbonate-alkalinity water are presented in Figure 1. For females, the HSI and CF in IP were significantly higher than those of SIRW; however, significantly lower GSI and TEY in IP were observed (*p* < 0.05, Figure 1A,C). For males, the GSI, MY, and TEY of SIRW were significantly higher than those of IP (*p* < 0.05, Figure 1B).

### 3.2. Color Parameters

The color parameters of adult *E. sinensis* reared in carbonate-alkalinity water are shown in Table 1. A significantly higher L* value of male dried carapace was observed in IP than that in SIRW (*p* < 0.05). For hepatopancreas, there were extremely significantly different b* values of female *E. sinensis* between IP and SIRW (*p* < 0.01), while significant differences were also observed by the a* value of female wet hepatopancreas, the L* value of male wet hepatopancreas, and the b* values of male hepatopancreas (*p* < 0.05). No significant differences were found in ovary color between IP and SIRW (*p* > 0.05).

### 3.3. Proximate Composition

The proximate compositions of adult *E. sinensis* reared in carbonate-alkalinity water are presented in Table 2. The contents of crude protein and crude fat in the female hepatopancreas in IP were significantly increased compared with that in SIRW (*p* < 0.05). The male gonad crude protein content of *E. sinensis* from SIRW was significantly higher than that in IP (*p* < 0.05), while the content of crude protein in male muscle in IP was lower than that in SIRW, with an extremely significant difference (*p* < 0.01).

### 3.4. Fatty Acids Profiles

Table 3 illustrates the evaluation and comparison of the main fatty acid composition, concentration, h/H, AI, and TI of adult *E. sinensis* reared in carbonate-alkalinity water. For females, higher concentrations of ∑SFA, ∑n−3 PUFA, ∑LC-PUFA, and ∑DHA + EPA in all the edible tissues, and ∑PUFA in the ovary and muscle, were observed in SIRW samples than those in IP, with no significant difference (*p* > 0.05). Concentrations of ∑MUFA, ∑EFA, and h/H in all the edible tissues were observed to be higher in IP than those in SIRW, with the following significant differences: ∑MUFA in the hepatopancreas and ovary, ∑EFA in the muscle, and h/H in the ovary (*p* < 0.05). For males, the levels of ΣSFA in gonads and muscle, ΣPUFA in gonads, Σn−3 PUFA in muscle, and ΣDHA + EPA in hepatopancreas of SIRW-reared crabs were significantly higher than those in IP-reared crabs (*p* < 0.05). The concentrations of ∑MUFA, h/H in all the edible tissues, and ∑EFA in the hepatopancreas and muscle detected were higher in IP than those in SIRW, with the following significant differences: ∑MUFA, ∑EFA of the hepatopancreas between IP and SIRW (*p* < 0.05), ∑MUFA of the gonad system and muscle, and ∑EFA of muscle between IP and SIRW (*p* < 0.01).

### 3.5. Free Amino Acids Composition and Taste Activity Value

The composition and contents of free amino acids (FAAs) in adult *E. sinensis* reared in carbonate-alkalinity water are presented in Table 4. With respect to females, the concentrations of serine (Ser), isoleucine (Ile), lysine (Lys), valine (Val), and ∑EFAA in the hepatopancreas were significantly higher in IP crabs than in SIRW-reared crabs (*p* < 0.05); however, the concentrations of alanine (Ala), histidine (His), Lys, and methionine (Met) observed in the muscle were higher in SIRW compared with those in IP (*p* < 0.05). For males, significant differences were noted by observing Ile in the gonad system and ∑FAA in the muscle (*p* < 0.05), while extremely significant differences were also observed in Ala in the hepatopancreas and muscle, and in glycine (Gly) in the muscle between (*p* < 0.01).

The flavor characteristics and taste activity values (TAV) of adult *E. sinensis* reared in carbonate-alkalinity water are shown in Table 5. The 17 FAAs were separated into two tastes including pleasant taste (umami and sweetness) and unpleasant taste (bitterness). The concentrations of ∑TUV and ∑TBV in the female hepatopancreas of IP were higher than those in SIRW; however, higher concentrations of ∑TSV were observed in SIRW samples. Higher concentrations of ∑TSV and ∑TBV in the ovary were detected in SIRW samples than in IP. In the muscle and male hepatopancreas, the ∑TUV, ∑TSV, and ∑TBV values of IP were lower than those of SIRW.

### 3.6. Mineral Element Composition

The mineral element composition and contents of adult *E. sinensis* reared in carbonate-alkalinity water are presented in Table 6. Regarding the hepatopancreas, there was no significant difference observed between IP and SIRW (*p* > 0.05). The levels of the K, Ca, Mg, Fe, and Zn elements in the ovary of IP-reared crabs were significantly higher than in SIRW crabs (*p* < 0.05); meanwhile, the content of ∑TME in IP was extremely significantly different compared with that in SIRW (*p* < 0.01). The contents of K and Mg in female muscle, as well as Na and ΣTME in male muscle, in IP crabs were significantly higher than in SIRW crabs (*p* < 0.05). Overall, higher contents of hepatopancreas ∑TME were observed in SIRW; however, the contents of gonad and muscle ∑TME in IP were higher than those in SIRW.

## 4. Discussion

### 4.1. Total Edible Yield

The hepatopancreas, gonad, and muscle are important edible tissues of *E. sinensis*. However, previous studies have focused on the total edible yield of *E. sinensis* reared in freshwater- [26,27], chloride-, or sulfate-type saline–alkaline water [28], while little attention has been paid to *E. sinensis* reared in carbonate-alkalinity water. The present study indicates that *E. sinensis* can complete gonadal development normally compared with previous studies [5,22]. The GSI value of female *E. sinensis* reared in IP was 3.78 ± 0.67% on 4th September and 6.75 ± 0.55% in SRW on 29th September, while the GSI value of female *E. sinensis* in freshwater pond was 5.30 ± 0.22% on 15th September [22]. It has been demonstrated that the later the sampling period, the usually higher the GSI in the same location, which is consistent with the present study. The specific values between previous and present studies were different, which was mainly caused by the difference in water temperature and sampling period [5,22,26,27]. The MY and TEY values of female *E. sinensis* in the present study were 26.11 ± 0.67%, 26.53 ± 0.28%, 38.19 ± 0.98%, and 40.78 ± 0.68%, respectively, indicating that *E. sinensis* reared in carbonate-alkalinity water have similar MY and TEY values compared with those obtained in previous studies [5,26,27]. Overall, the above results show that rearing of *E. sinensis* in carbonate-alkalinity water will not significantly affect the gonadal development and the total edible yield.

### 4.2. Color Parameters

Color is one of the important indicators of sensory and quality in evaluation of *E. sinensis*. In the cognition of consumers, the higher reddish values of the dried carapace and ovary, as well as the higher reddish and yellowish values of the wet hepatopancreas, suggest better quality [21]. It has been demonstrated that the reddish and yellowish parameters of *E. sinensis* tissues are significantly related to the deposition of carotenoids [29]. The present study illustrates that the b* values of IP [female: 40.99 ± 1.14; male: 45.85 ± 1.10] and SIRW [female: 42.19 ± 1.15; male: 43.29 ± 0.88] freeze-dried *E. sinensis* carapace reared in carbonate-alkalinity water were obviously higher than those of wild-caught mitten crabs in the natural Suifenhe and Nanliujinag delta [30], suggesting that *E. sinensis* reared in carbonate-alkalinity water accumulated more carotenoids. Similar b* values of freeze-dried three-year-old *E. sinensis* carapace were also observed in the pond of Zhaodong city, Heilongjiang Province, China [22]. In addition, the higher b* values in the ovary of IP [52.01 ± 0.19] and SIRW [51.34 ± 0.87] freeze-dried *E. sinensis* in the present study were also detected in carbonate-alkalinity water compared with previous studies [22,30]. These results suggest that *E. sinensis* reared in carbonate-alkalinity water accumulated more carotenoids and exhibited better quality.

### 4.3. Biochemical Composition

Biochemical composition, especially crude protein, has become one of the most important indicators for evaluating the nutritional value of aquatic animals, and can be affected by numerous factors, such as the culture environment [22]. The present study demonstrated that although the culture environment of IP and SIRW both belonged to carbonate-alkalinity water, significant differences were still observed between IP and SIRW. The crude protein in the female hepatopancreas from IP [11.17 ± 0.16%] and SIRW [9.63 ± 0.10%] was higher than those cultured in the Shandong, Qinghai, and Shanghai [26,28], as well as wild mitten crabs caught in Suifenhe, Liaohe, and Nanliujiang [30]. At the same time, the crude protein of gonad and muscle from *E. sinensis* reared in carbonate-alkalinity water were similar to those obtained in the previous studies [22,26,28,30]. All these results illustrate that *E. sinensis* reared in carbonate-alkalinity water is a good high-protein seafood source.

### 4.4. Fatty Acids Composition

Fatty acid composition and contents are also one of the most important nutritional indicators for aquatic animals, particularly essential fatty acids (EFAs) and unsaturated fatty acids (UFAs) [22,30]. The present study showed that the total saturated fatty acids (∑SFA), total monounsaturated fatty acids (∑MUFA), and total polyunsaturated fatty acids (∑PUFA) of *E. sinensis* reared in carbonate-alkalinity water were similar to those cultured in the other regions [22,27,28,30], implying that water alkalinity did not significantly alter the fatty acid composition of *E. sinensis*. It can be estimated that the differential fatty acid composition and content changes in the edible tissues of *E. sinensis* are mainly a result of heredity, followed by culture environment and diet. Culture environment can slightly regulate fatty acid contents other than that of composition.

Due to the fact that PUFAs are more beneficial to human health, they have received widespread attention, especially DHA, EPA, and ARA [30]. Numerous studies have confirmed that DHA and EPA can play an important role in preventing inflammation and cardiovascular diseases, while ARA can promote the development of the central nervous system [31,32,33]. The present study demonstrated that water alkalinity affected the fatty acid contents of *E. sinensis* compared with previous studies [26,27]. Furthermore, increasing DHA, EPA, and ARA contents in males, but decreasing DHA and EPA contents in females, were detected, implying the differential effects of alkalinity on each gender of *E. sinensis*. These results are consistent with our previous studies [5]. As *E. sinensis* were reared in similar carbonate-alkalinity water but with a different culture type, the EPA and ARA contents of male hepatopancreas and muscle in SIRW were significantly increased compared with those in IP. This phenomenon can be explained by the different developmental stages because the EPA and ARA contents of the hepatopancreas and muscle increased with the GSI improvement [27]. The h/H, AI, and TI are important indexes used to evaluate the beneficial effects of fatty acids on human health [22,25]. Generally, higher h/H, and the lower AI and TI are illustrated to be of better quality for human health. The three indexes of *E. sinensis* reared in carbonate-alkalinity water were similar to the results of previous studies [22,30].

### 4.5. FAAs Composition and TAV Analysis

The FAAs composition and contents are important factors affecting the taste and quality of crustaceans [22]. The present study illustrated that the ∑EFAA [IP: (724.11 ± 14.76) mg/100 g; SIRW: (632.4 ± 5.94) mg/100 g] and ∑FAA [IP: (1937.29 ± 85.06) mg/100 g; SIRW: (1788.67 ± 146.25) mg/100 g] contents of female *E. sinensis* hepatopancreas reared in carbonate-alkalinity water were higher than wild-caught mitten crabs [30] and three-year-old *E. sinensis* [22], indicating better taste quality. These results were consistent with our previous studies, in which prolonged alkalinity stress can improve ∑EFAA and ∑FAA contents [5]. In terms of specific amino acids, differential amino acids present different taste characteristics, while the taste characteristic, TAV, is positively correlated with the ratio between the special amino acid value and its threshold. Table 5 shows that the main umami amino acid Glu and the sweetness amino acid Ala were similar to previous studies [22,28,30]. Although we have classified Arg as a bitter amino acid, the flavor characteristic of Arg is actually significantly related to its concentration, with low concentrations exhibiting bitterness and high concentrations exhibiting umami [34]. The present study illustrates that the Arg content [IP: 7.42; SIRW: 6.50] in the female hepatopancreas was slightly higher than that of previous studies [5,22,30]. In addition, the ∑TUV, ∑TSV, and ∑TBV values of female hepatopancreas were higher than those in the pond-reared and wild-caught mitten crabs [22,30], suggesting stronger flavor characteristics.

### 4.6. Mineral Element Analysis

Mineral elements are important nutritional substances required to maintain normal growth, development, and metabolism in human beings, ensuring normal life activities [35]. Na, K, Ca, and Mg are macro-elements required by the human body, and they play differential roles in human health. Among these four macro-elements, Na and K play an important role in maintaining the acid–base balance and osmotic pressure of blood and body fluids. Ca is an important component of human bones and teeth. Mg participates in energy metabolism in the human body, catalyzes and activates various enzyme systems, and plays an important role in preventing cardiovascular diseases [36]. Of all the *E. sinensis* edible tissues, Na and K contents were significantly higher than other macro-elements, which was consistent with previous studies [22,37]. In addition, the K content was higher than the Na content except for the testis, implying that the culture environment perhaps has influence on Na and K accumulation. Fe, Zn, Cu, and Mn are micro-elements required by the human body. Among these, Fe plays an important role in the body’s hematopoietic, oxygen transport, and fluid balance. Zn is an important coenzyme factor in the human body and is involved in the synthesis of DNA, RNA, and proteins. Cu can promote the production of hemoglobin in humans. Mn plays an important role in the central nervous system of the human brain [37]. A higher Fe content in the *E. sinensis* hepatopancreas was observed compared with that in the gonad and muscle tissues; furthermore, Fe is an important source for humans and has a certain significance in preventing iron deficiency anemia. Higher Zn contents were noted in the *E. sinensis* ovary and muscle compared with those in the testis and hepatopancreas tissues. The above results illustrate that *E. sinensis* reared in carbonate-alkalinity water is a good source of mineral elements.

## 5. Conclusions

The present study investigated the gonadal development, edible yield, coloration, nutritional, and flavor quality of *E. sinensis* reared in carbonate-alkalinity water (IP and SIRW culture model). Due to the sampling time, differential GSI and TEY values of *E. sinensis* were observed between IP and SIRW. IP samples accumulated more ∑MUFA, ∑EFA, and h/H in the female edible tissues compared with those of SIRW, whereas SIRW had better ∑FAA content in the muscle. IP samples also exhibited higher mineral elements, such as K, Ca, Mg, Fe, and Zn in the ovary. In summary, *E. sinensis* reared in carbonate-alkalinity water can complete gonadal development, accumulate more carotenoids, and are rich in fatty acids, FFAs, and mineral elements.

## Figures and Tables

**Table 1:** The color comparison of adult *Eriocheir sinensis* reared in carbonate-alkalinity water.

Item		Color	Female		Male	
			IP	SIRW	IP	SIRW
Carapace	Wet sample	L*	47.18 ± 0.99	48.57 ± 0.95	47.78 ± 0.70	49.51 ± 1.13
		a*	2.76 ± 0.29	2.78 ± 0.21	3.26 ± 0.24	3.26 ± 0.40
		b*	15.04 ± 0.39	16.59 ± 0.77	16.08 ± 0.48	17.70 ± 0.62
	Dried sample	L*	67.09 ± 1.15	64.89 ± 0.95	70.16 ± 1.00 *	65.80 ± 1.11
		a*	21.30 ± 0.96	21.29 ± 1.02	20.79 ± 1.12	20.95 ± 1.53
		b*	40.99 ± 1.14	42.19 ± 1.15	45.85 ± 1.10	43.29 ± 0.88
Hepatopancreas	Wet sample	L*	62.22 ± 1.61	61.52 ± 1.25	54.65 ± 2.18	62.46 ± 2.18 *
		a*	15.52 ± 1.89	21.65 ± 1.31 *	16.90 ± 1.13	16.59 ± 1.33
		b*	43.38 ± 2.94	54.23 ± 1.32 **	39.46 ± 2.42	48.41 ± 2.48 *
	Dried sample	L*	60.60 ± 2.73	63.99 ± 2.12	57.31 ± 4.01	64.41 ± 1.43
		a*	12.53 ± 3.25	15.43 ± 1.42	14.01 ± 1.48	13.22 ± 0.92
		b*	37.49 ± 1.49	48.43 ± 1.26 **	35.17 ± 1.31	45.26 ± 2.37 *
Gonad	Wet sample	L*	28.55 ± 0.48	28.25 ± 0.32	——	——
		a*	1.78 ± 0.67	1.22 ± 0.27	——	——
		b*	3.96 ± 0.41	3.88 ± 0.24	——	——
	Dried sample	L*	74.96 ± 1.77	73.96 ± 0.54	——	——
		a*	30.93 ± 2.00	32.31 ± 0.86	——	——
		b*	52.01 ± 0.19	51.34 ± 0.87	——	——

Note: Data are presented as means ± standard error (SE) (n = 25). “——” means no detection. * denotes significant difference (*p* < 0.05), ** denotes extreme significant difference (*p* < 0.01). Abbreviations: IP, *Eriocheir sinensis* reared in intensive pond; SIRW, *Eriocheir sinensis* reared in semi-intensive reed wetland.

**Table 2:** The proximate composition of adult *Eriocheir sinensis* reared in carbonate-alkalinity water (%, wet weight).

Item	Female		Male	
	IP	SIRW	IP	SIRW
Hepatopancreas				
Moisture	48.74 ± 5.16	61.72 ± 3.17	61.18 ± 1.82	62.32 ± 1.69
Crude protein	11.17 ± 0.16 *	9.63 ± 0.10	8.79 ± 1.46	8.51 ± 1.03
Crude fa	35.39 ± 0.05 *	23.79 ± 1.21	25.21 ± 1.88	25.11 ± 1.45
Ash	1.31 ± 0.08	1.57 ± 0.27	1.09 ± 0.19	1.17 ± 0.19
Gonad				
Moisture	56.46 ± 2.89	55.25 ± 2.66	76.08 ± 0.62	75.05 ± 0.77
Crude protein	28.90 ± 0.23	28.83 ± 0.10	15.90 ± 0.06	16.62 ± 0.09 *
Crude fat	6.42 ± 0.50	7.23 ± 0.02	0.72 ± 0.07 *	0.37 ± 0.02
Ash	1.89 ± 0.11	1.83 ± 0.04	2.08 ± 0.05	2.20 ± 0.05
Muscle				
Moisture	78.63 ± 0.67	79.73 ± 0.55	83.69 ± 0.56	82.29 ± 0.35
Crude protein	17.76 ± 0.16	17.46 ± 0.03	13.64 ± 0.06	15.07 ± 0.06 **
Crude fat	0.50 ± 0.03	0.42 ± 0.03	0.44 ± 0	0.39 ± 0.02
Ash	1.44 ± 0.03	1.54 ± 0.02	1.38 ± 0.01	1.48 ± 0.08

Notes: Data are presented as means ± standard error (SE) (n = 3). * denotes significant difference (*p* < 0.05), ** denotes extreme significant difference (*p* < 0.01). Abbreviations: IP, *Eriocheir sinensis* reared in intensive pond; SIRW, *Eriocheir sinensis* reared in semi-intensive reed wetland.

**Table 3:** The fatty acid composition in the hepatopancreas, gonad, and muscle of adult *Eriocheir sinensis* reared in carbonate-alkalinity water (% of total fatty acids).

Fatty Acid	Hepatopancreas		Gonad		Muscle	
	IP	SIRW	IP	SIRW	IP	SIRW
Female						
C14:0	1.51 ± 0.17	2.17 ± 0.47	0.79 ± 0.03	1.20 ± 0.08 *	0.33 ± 0.05	0.51 ± 0.05
C15:0	0.63 ± 0.03	1.31 ± 0.34	0.47 ± 0.06	0.93 ± 0.10	0.27 ± 0.02	0.47 ± 0.04 *
C16:0	22.26 ± 0.73	23.01 ± 1.24	16.59 ± 0.39	17.73 ± 0.59	12.96 ± 0.40	13.73 ± 0.51
C17:0	0.61 ± 0.01	1.34 ± 0.14 *	0.50 ± 0.06	0.77 ± 0.04	0.73 ± 0.07	1.34 ± 0.14
C18:0	3.14 ± 0.13	3.72 ± 0.34	2.72 ± 0.09	3.05 ± 0.14	8.98 ± 0.01	9.33 ± 0.20
C20:0	0.32 ± 0.02	0.41 ± 0.03	0.09 ± 0.01	0.13 ± 0	0.17 ± 0.02	0.28 ± 0.01 *
∑SFA	29.24 ± 1.11	33.24 ± 2.72	21.25 ± 0.63	24.06 ± 0.47	23.42 ± 0.57	25.65 ± 0.54
C15:1n5	0.23 ± 0.02	0.45 ± 0.10	0.15 ± 0	0.26 ± 0.02 *	0.85 ± 0.09	0.78 ± 0.06
C16:1n7	8.38 ± 0.30	10.95 ± 0.34 *	11.40 ± 0.01	13.65 ± 1.04	3.04 ± 0.21	4.26 ± 0.18 *
C18:1n9	39.15 ± 0.15 **	32.45 ± 0.40	33.69 ± 1.13 *	26.10 ± 1.12	25.92 ± 0.65	22.20 ± 0.63
C20:1n9	1.07 ± 0.03	1.28 ± 0.10	0.45 ± 0.02	0.34 ± 0.03	0.71 ± 0.07	0.81 ± 0.09
∑MUFA	49.42 ± 0.43 *	45.89 ± 0.27	46.07 ± 1.09 *	40.51 ± 0.07	30.51 ± 1.01	28.04 ± 0.48
C18:2n6	15.81 ± 0.20 *	11.56 ± 0.78	17.19 ± 0.80	13.87 ± 0.84	13.46 ± 0.87 *	8.71 ± 0.09
C18:3n3	2.17 ± 0.04	2.93 ± 0.51	3.78 ± 0.58	6.21 ± 1.39	1.89 ± 0.05	2.73 ± 0.14 *
C20:2n6	0.95 ± 0.26	0.88 ± 0.02	0.95 ± 0.06	0.81 ± 0.05	1.50 ± 0.11	1.47 ± 0.06
C20:4n6 (ARA)	0.65 ± 0.10	1.55 ± 0.43	2.33 ± 0.19	3.85 ± 0.18 *	5.40 ± 0.42	8.14 ± 0.47 *
C20:3n3	0.24 ± 0.06	0.48 ± 0.08	0.38 ± 0.03	0.55 ± 0.08	0.43 ± 0.03	0.74 ± 0.05 *
C20:5n3 (EPA)	0.77 ± 0.12	1.61 ± 0.60	4.72 ± 0.53	6.68 ± 0.12	14.05 ± 0.19	16.21 ± 1.16
C22:6n3 (DHA)	0.48 ± 0.04	1.28 ± 0.62	3.20 ± 0.10	2.82 ± 0.01	9.18 ± 0.86	7.92 ± 0
∑PUFA	21.29 ± 0.72	20.89 ± 2.98	32.85 ± 0.51	35.34 ± 0.53	46.08 ± 1.57	46.67 ± 0.65
∑EFA	17.97 ± 0.24	14.48 ± 1.28	20.97 ± 0.22	20.07 ± 0.55	15.35 ± 0.81 *	11.44 ± 0.05
∑LC-PUFA	3.32 ± 0.48	6.21 ± 1.71	11.71 ± 0.81	14.99 ± 0.04	30.55 ± 0.77	34.74 ± 0.63
∑n−3 PUFA	3.65 ± 0.18	6.29 ± 1.27	12.08 ± 0.87	16.26 ± 1.11	25.54 ± 0.72	27.59 ± 0.95
∑n−6 PUFA	17.65 ± 0.27	14.60 ± 0.68	20.78 ± 0.42	19.09 ± 0.60	20.55 ± 0.31	19.08 ± 0.40
n−3/n−6 PUFA	0.21 ± 0.01	0.42 ± 0.09	0.58 ± 0.08	0.86 ± 0.13	1.24 ± 0.02	1.45 ± 0.12
∑DHA + EPA	1.25 ± 0.15	2.89 ± 1.22	7.92 ± 0.62	9.50 ± 0.11	23.22 ± 1.05	24.13 ± 1.16
DHA/EPA	0.63 ± 0.04	0.76 ± 0.11	0.68 ± 0.06 *	0.42 ± 0.01	0.65 ± 0.05	0.49 ± 0.04
h/H	2.48 ± 0.12	2.06 ± 0.27	3.72 ± 0.12 *	3.15 ± 0.05	5.23 ± 0.23	4.65 ± 0.18
AI	0.40 ± 0.03	0.48 ± 0.07	0.25 ± 0.01	0.30 ± 0.01	0.19 ± 0.01	0.21 ± 0.01
TI	0.60 ± 0.04	0.60 ± 0.11	0.29 ± 0	0.28 ± 0.02	0.22 ± 0.01	0.22 ± 0.01
Male						
C14:0	1.35 ± 0.16	1.59 ± 0.22	0.37 ± 0.03	0.48 ± 0.04	0.26 ± 0.01	0.36 ± 0.01 **
C15:0	0.70 ± 0.05	0.89 ± 0.13	0.28 ± 0.07	0.30 ± 0.02	0.25 ± 0.01	0.43 ± 0.03 *
C16:0	22.16 ± 0.12	21.49 ± 1.23	9.26 ± 0.27	10.43 ± 0.33	12.93 ± 0.11	13.18 ± 0.49
C17:0	0.53 ± 0.05	0.94 ± 0.02 *	0.62 ± 0.02	0.92 ± 0.10	0.76 ± 0.03	1.33 ± 0 **
C18:0	3.37 ± 0.01	3.26 ± 0.10	8.06 ± 0.05	7.92 ± 0.26	9.04 ± 0.18	9.90 ± 0.06 *
C20:0	0.37 ± 0	0.34 ± 0.02	0.25 ± 0	0.26 ± 0.01	0.17 ± 0.01	0.26 ± 0 **
∑SFA	29.52 ± 0.33	29.53 ± 0.88	19.32 ± 0.32	20.70 ± 0.02 *	23.39 ± 0.11	25.45 ± 0.46 *
C15:1n5	0.25 ± 0.02	0.24 ± 0.13	0.93 ± 0.05	0.73 ± 0.08	1.08 ± 0.03	1.02 ± 0.02
C16:1n7	7.79 ± 0.99	10.79 ± 1.18	2.03 ± 0.27	2.31 ± 0.10	2.03 ± 0.11	2.64 ± 0.04 *
C18:1n9	36.24 ± 0.77 *	31.27 ± 0.62	23.88 ± 0.24	23.19 ± 0.17	25.31 ± 0.07 **	20.52 ± 0.16
C20:1n9	1.09 ± 0.05	1.01 ± 0.04	1.34 ± 0.04	1.22 ± 0.10	0.79 ± 0.01	0.79 ± 0.07
∑MUFA	46.03 ± 0.21 *	43.86 ± 0.43	35.91 ± 0.02 **	30.20 ± 0.43	29.20 ± 0.06 **	24.96 ± 0.25
C18:2n6	19.19 ± 0.04 *	14.51 ± 0.79	9.84 ± 0.79	10.13 ± 3.01	12.10 ± 0.21 *	8.59 ± 0.35
C18:3n3	1.98 ± 0.07	3.35 ± 0.89	1.00 ± 0.09	1.60 ± 0.17	1.40 ± 0.04	2.10 ± 0.27
C20:2n6	0.76 ± 0.19	0.86 ± 0.04	3.17 ± 0.11 *	2.34 ± 0.08	1.80 ± 0.01	1.61 ± 0.11
C20:4n6 (ARA)	0.65 ± 0.11	2.11 ± 0.28 *	11.78 ± 0.63	15.16 ± 0.52	6.92 ± 0.15	9.96 ± 0.70 *
C20:3n3	0.17 ± 0.03	0.52 ± 0.16	0.61 ± 0.02	0.80 ± 0.09	0.44 ± 0.01	0.70 ± 0.03 **
C20:5n3 (EPA)	0.83 ± 0.08	2.20 ± 0.23 *	11.13 ± 0.10	12.35 ± 1.31	14.74 ± 0.09	16.18 ± 0.02 **
C22:6n3 (DHA)	0.67 ± 0.23	2.38 ± 0.41	6.81 ± 0.37	5.98 ± 0.72	9.82 ± 0.09	9.85 ± 0.58
∑PUFA	24.45 ± 0.54	26.62 ± 1.31	44.78 ± 0.31	49.10 ± 0.45 *	47.41 ± 0.06	49.59 ± 0.72
∑EFA	21.17 ± 0.11 **	17.86 ± 0.11	10.84 ± 0.88	11.73 ± 3.18	13.50 ± 0.17 *	10.67 ± 0.62
∑LC-PUFA	3.29 ± 0.64	8.59 ± 1.21	33.70 ± 1.19	36.93 ± 2.73	33.71 ± 0.13	38.56 ± 1.41
∑n−3 PUFA	3.65 ± 0.19	8.44 ± 1.20	19.55 ± 0.28	20.72 ± 1.37	26.39 ± 0.03	28.82 ± 0.30 *
∑n−6 PUFA	20.80 ± 0.16 *	18.18 ± 0.22	25.23 ± 0.05	28.38 ± 1.38	21.02 ± 0.05	20.78 ± 0.43
n−3/n−6 PUFA	0.18 ± 0.01	0.47 ± 0.10	0.78 ± 0.02	0.74 ± 0.13	1.26 ± 0.01	1.39 ± 0.01 *
∑DHA + EPA	1.50 ± 0.31	4.58 ± 0.65 *	17.94 ± 0.47	18.32 ± 2.02	24.56 ± 0	26.03 ± 0.59
DHA/EPA	0.79 ± 0.20	1.08 ± 0.07	0.61 ± 0.03	0.48 ± 0.01	0.67 ± 0.01	0.61 ± 0.03
h/H	2.52 ± 0.08	2.44 ± 0.18	6.65 ± 0.21	6.26 ± 0.13	5.30 ± 0.04	4.97 ± 0.21
AI	0.39 ± 0.01	0.40 ± 0.01	0.13 ± 0.01	0.16 ± 0	0.18 ± 0	0.20 ± 0.01
TI	0.60 ± 0.02	0.47 ± 0.06	0.20 ± 0.01	0.20 ± 0.01	0.21 ± 0	0.21 ± 0.01

Notes: Data are presented as means ± standard error (SE) (n = 3). * denotes significant difference (*p* < 0.05). ** denotes extreme significant difference (*p* < 0.01). Abbreviations: IP, *Eriocheir sinensis* reared in intensive pond; SIRW, *Eriocheir sinensis* reared in semi-intensive reed wetland;∑SFA, total saturated fatty acids; ∑MUFA, total monounsaturated fatty acids; ∑PUFA, total polyunsaturated fatty acids; ∑EFA, total essential fatty acids; ∑LC-PUFA, total long chain polyunsaturated fatty acids; ∑n−3 PUFA, total ω-3 polyunsaturated fatty acids; ∑n−6 PUFA, total ω-6 polyunsaturated fatty acids; DHA, docosahesaenoic acid; EPA, eicosapentaenoic acid, ARA, arachidonic acid; h/H, hypocholesterolaemic/hypercholesterolaemic ratio; AI, index of atherogenicity; TI, index of thrombogenicity.

**Table 4:** The free amino acid composition in the hepatopancreas, gonad, and muscle of adult *Eriocheir sinensis* reared in carbonate-alkalinity water (mg/100 g, wet weight).

Free Amino Acids	Hepatopancreas		Gonad		Muscle	
	IP	SIRW	IP	SIRW	IP	SIRW
Female						
Aspartic acid	61.64 ± 1.28	41.42 ± 6.26	2.80 ± 0.54	3.45 ± 0.50	2.18 ± 0.27	2.94 ± 0.31
Arginine	370.76 ± 18.22	324.80 ± 57.25	222.08 ± 11.32	274.41 ± 6.80	556.48 ± 11.41	548.28 ± 35.14
Alanine	233.89 ± 25.35	285.79 ± 53.52	100.10 ± 10.94	137.58 ± 3.70	338.37 ± 8.75	530.03 ± 25.96 *
Cysteine	17.16 ± 0.92	14.76 ± 2.11	2.68 ± 0.76	2.73 ± 0.27	2.17 ± 0.18	1.68 ± 0
Glutamic acid	135.80 ± 11.69	113.99 ± 0.14	97.72 ± 7.99	86.44 ± 5.72	33.63 ± 3.46	46.17 ± 14.28
Glycine	130.66 ± 21.38	133.69 ± 24.05	60.24 ± 5.88	64.19 ± 10.08	347.70 ± 24.21	453.18 ± 78.36
Histidine	43.34 ± 7.62	39.31 ± 4.41	27.93 ± 4.53	32.22 ± 5.14	22.88 ± 2.42	35.61 ± 1.30 *
Proline	92.75 ± 16.01	107.32 ± 23.94	71.14 ± 5.25	73.86 ± 2.83	135.70 ± 7.48	147.75 ± 23.28
Serine	28.33 ± 0.66 *	21.30 ± 0.68	8.21 ± 0.55	9.60 ± 1.23	9.94 ± 1.17	12.84 ± 1.79
Tyrosine	98.84 ± 8.62	73.88 ± 3.42	18.61 ± 1.70	19.23 ± 1.67	18.08 ± 2.52	20.06 ± 2.60
Isoleucine ^▲^	67.41 ± 0.04 *	60.24 ± 1.08	9.32 ± 1.38	10.26 ± 2.22	8.61 ± 0.03	13.76 ± 3.10
Leucine ^▲^	156.88 ± 3.55	130.35 ± 6.04	13.60 ± 1.94	14.58 ± 2.94	15.91 ± 0.94	27.06 ± 6.37
Lysine ^▲^	155.55 ± 1.00 *	138.35 ± 3.31	38.97 ± 9.97	43.95 ± 11.31	28.99 ± 0.07	39.34 ± 0.96 **
Methionine ^▲^	50.09 ± 0.52	51.19 ± 1.91	22.30 ± 2.65	22.31 ± 5.50	24.28 ± 2.51	43.56 ± 3.02 *
Phenylalanine ^▲^	92.75 ± 4.09	74.59 ± 2.04	15.49 ± 2.05	15.18 ± 2.40	11.57 ± 1.02	14.83 ± 1.23
Threonine ^▲^	94.83 ± 5.28	85.27 ± 7.19	66.20 ± 6.70	94.49 ± 5.91	25.18 ± 0	32.52 ± 10.71
Valine ^▲^	106.61 ± 2.37 *	92.42 ± 1.26	26.09 ± 3.65	28.94 ± 4.53	21.70 ± 0.40	31.46 ± 5.97
∑EFAA	724.11 ± 14.76 *	632.4 ± 5.94	191.98 ± 28.34	229.71 ± 34.81	136.24 ± 2.23	202.54 ± 31.35
∑FAA	1937.29 ± 85.06	1788.67 ± 146.25	803.48 ± 77.8	933.42 ± 45.92	1603.38 ± 9.64	2001.07 ± 162.46
PETFAA	37.42 ± 0.89	35.62 ± 3.24	23.77 ± 1.22	24.49 ± 2.52	8.50 ± 0.19	10.06 ± 0.75
Male						
Aspartic acid	43.85 ± 3.26	48.34 ± 24.86	24.44 ± 3.22	28.43 ± 5.07	2.27 ± 0.56	2.74 ± 0.04
Arginine	188.15 ± 29.12	266.95 ± 79.80	60.46 ± 5.69	47.63 ± 7.49	408.25 ± 8.95	451.24 ± 24.15
Alanine	139.45 ± 13.65	318.65 ± 0.04 **	97.27 ± 5.28	100.84 ± 10.70	331.50 ± 8.40	471.54 ± 7.67 **
Cysteine	8.73 ± 1.51	16.25 ± 8.61	1.44 ± 0.14	2.31 ± 0.58	3.06 ± 0.07 *	1.83 ± 0.12
Glutamic acid	94.93 ± 3.21	114.46 ± 46.49	68.52 ± 0.56	49.61 ± 5.25	53.15 ± 0.61	53.69 ± 1.81
Glycine	79.84 ± 1.00	135.22 ± 48.32	43.29 ± 6.29	42.69 ± 3.20	321.59 ± 12.02	465.93 ± 6.64 **
Histidine	30.11 ± 0.21	41.39 ± 11.40	8.26 ± 1.31	6.97 ± 0.41	22.76 ± 1.25	25.80 ± 1.42
Proline	66.41 ± 2.52	93.25 ± 8.48	61.24 ± 4.80	41.13 ± 1.57	97.51 ± 6.49	121.40 ± 22.25
Serine	17.96 ± 1.13	22.84 ± 7.64	3.11 ± 0.07 *	2.30 ± 0.14	6.54 ± 0.84	7.81 ± 1.76
Tyrosine	70.26 ± 1.48	83.70 ± 39.02	17.70 ± 0.46	18.37 ± 1.92	26.17 ± 4.63	23.25 ± 1.65
Isoleucine ^▲^	37.64 ± 2.23	64.30 ± 29.24	7.64 ± 0.37	11.55 ± 0.52 *	14.99 ± 2.55	12.50 ± 0.52
Leucine ^▲^	99.65 ± 11.15	138.90 ± 64.09	11.01 ± 0.50	13.65 ± 1.10	24.63 ± 3.85	23.31 ± 0.68
Lysine ^▲^	91.37 ± 13.89	145.85 ± 67.58	13.49 ± 2.08	12.36 ± 1.65	41.75 ± 4.79	30.91 ± 2.93
Methionine ^▲^	36.67 ± 2.62	53.00 ± 23.41	7.50 ± 1.90	11.32 ± 0.43	31.16 ± 3.14	32.16 ± 3.17
Phenylalanine ^▲^	60.00 ± 2.35	81.29 ± 37.07	10.27 ± 0.29	13.43 ± 1.35	14.21 ± 1.94	16.85 ± 1.25
Threonine ^▲^	57.42 ± 2.41	85.36 ± 25.80	15.66 ± 2.54	10.54 ± 0.89	35.47 ± 2.69	27.29 ± 2.66
Valine ^▲^	60.49 ± 5.91	94.62 ± 36.67	17.26 ± 0.96	17.16 ± 1.50	34.95 ± 1.08	30.06 ± 0.48
∑EFAA	443.23 ± 40.57	663.33 ± 283.85	82.82 ± 8.64	90.01 ± 7.45	197.17 ± 8.38	173.08 ± 5.84
∑FAA	1182.92 ± 95.24	1804.37 ± 558.45	468.56 ± 25.61	430.3 ± 43.77	1469.95 ± 5.52	1798.31 ± 56.21 *
PETFAA	37.44 ± 0.42	35.27 ± 4.81	17.63 ± 0.88	20.96 ± 0.40	13.42 ± 0.62 *	9.62 ± 0.02

Notes: Data are presented as means ± standard error (SE) (n = 3). ^▲^ essential amino acid. * denotes significant difference (*p* < 0.05). ** denotes extreme significant difference (*p* < 0.01). Abbreviations: IP, *Eriocheir sinensis* reared in intensive pond; SIRW, *Eriocheir sinensis* reared in semi-intensive reed wetland; ∑EFAA, total essential free amino acids; ∑FAA, total free amino acids; PETFAA, percentage of ∑EFAA to ∑FAA.

**Table 5:** The threshold and taste activity value of free amino acid composition in the hepatopancreas, gonad, and muscle of adult *Eriocheir sinensis* reared in carbonate-alkalinity water.

Free Amino Acids	Flavor Characteristics	Threshold (mg/100 mL)	Hepatopancreas		Gonad		Muscle	
			IP	SIRW	IP	SIRW	IP	SIRW
Female								
Aspartic acid	umami (+)	100	0.62	0.41	0.03	0.03	0.02	0.03
Glutamic acid	umami (+)	30	4.53	3.80	3.26	2.88	1.12	1.54
∑TUV			5.14	4.21	3.29	2.92	1.14	1.57
Alanine	sweetness (+)	60	3.90	4.76	1.67	2.29	5.64	8.83
Glycine	sweetness (+)	130	1.01	1.03	0.46	0.49	2.67	3.49
Serine	sweetness (+)	150	0.19	0.14	0.05	0.06	0.07	0.09
Threonine	sweetness (+)	260	0.36	0.33	0.25	0.36	0.10	0.13
Proline	sweetness/bitterness (+)	300	0.31	0.36	0.24	0.25	0.45	0.49
∑TSV			5.77	6.62	2.68	3.46	8.93	13.02
Arginine	sweetness/bitterness (−)	50	7.42	6.50	4.44	5.49	11.13	10.97
Lysine	sweetness/bitterness (−)	50	3.11	2.77	0.78	0.88	0.58	0.79
Valine	sweetness/bitterness (−)	40	2.67	2.31	0.65	0.72	0.54	0.79
Methionine	bitterness/sweetness/sulphur (−)	30	1.67	1.71	0.74	0.74	0.81	1.45
Histidine	bitterness (−)	20	2.17	1.97	1.40	1.61	1.14	1.78
Isoleucine	bitterness (−)	90	0.75	0.67	0.10	0.11	0.10	0.15
Leucine	bitterness (−)	190	0.83	0.69	0.07	0.08	0.08	0.14
Phenylalanine	bitterness (−)	90	1.03	0.83	0.17	0.17	0.13	0.16
∑TBV			19.60	17.40	8.40	9.80	14.50	16.20
Male								
Aspartic acid	umami (+)	100	0.44	0.48	0.24	0.28	0.02	0.03
Glutamic acid	umami (+)	30	3.16	3.82	2.28	1.65	1.77	1.79
∑TUV			3.60	4.30	2.53	1.94	1.79	1.82
Alanine	sweetness (+)	60	2.32	5.31	1.62	1.68	5.52	7.86
Glycine	sweetness (+)	130	0.61	1.04	0.33	0.33	2.47	3.58
Serine	sweetness (+)	150	0.12	0.15	0.02	0.02	0.04	0.05
Threonine	sweetness (+)	260	0.22	0.33	0.06	0.04	0.14	0.10
Proline	sweetness/bitterness (+)	300	0.22	0.31	0.20	0.14	0.33	0.40
∑TSV			3.50	7.14	2.24	2.20	8.50	12.00
Arginine	sweetness/bitterness (−)	50	3.76	5.34	1.21	0.95	8.17	9.02
Lysine	sweetness/bitterness (−)	50	1.83	2.92	0.27	0.25	0.84	0.62
Valine	sweetness/bitterness (−)	40	1.51	2.37	0.43	0.43	0.87	0.75
Methionine	bitterness/sweetness/sulphur (−)	30	1.22	1.77	0.25	0.38	1.04	1.07
Histidine	bitterness (−)	20	1.51	2.07	0.41	0.35	1.14	1.29
Isoleucine	bitterness (−)	90	0.42	0.71	0.08	0.13	0.17	0.14
Leucine	bitterness (−)	190	0.52	0.73	0.06	0.07	0.13	0.12
Phenylalanine	bitterness (−)	90	0.67	0.90	0.11	0.15	0.16	0.19
∑TBV			11.40	16.80	2.80	2.70	12.50	13.20

Note: + means pleasant taste; − means unpleasant taste. Abbreviations: IP, *Eriocheir sinensis* reared in intensive pond; SIRW, *Eriocheir sinensis* reared in semi-intensive reed wetland; ∑TUV, total umami values; ∑TSV, total sweetness values; ∑TBV, total bitterness values.

**Table 6:** The mineral element composition in the hepatopancreas, gonad, and muscle of adult *Eriocheir sinensis* reared in carbonate-alkalinity water (mg/kg, wet weight).

Element	Hepatopancreas		Gonad		Muscle	
	IP	SIRW	IP	SIRW	IP	SIRW
Female						
Na	1635.77 ± 148.67	1710.28 ± 312.14	2113.71 ± 440.18	1061.98 ± 78.54	3027.63 ± 495.63	2472.82 ± 56.59
K	2407.05 ± 82.20	2642.24 ± 599.61	2715.20 ± 201.54 *	2090.29 ± 72.20	3947.00 ± 129.33 *	3383.32 ± 118.73
Ca	488.47 ± 97.24	835.46 ± 147.10	487.54 ± 65.58 *	230.54 ± 26.95	1135.93 ± 93.36	894.56 ± 73.72
Mg	349.11 ± 22.89	367.95 ± 77.81	1514.93 ± 100.57 *	705.99 ± 102.58	696.91 ± 50.97 *	491.37 ± 11.33
Fe	79.48 ± 8.06	79.82 ± 9.65	41.61 ± 4.00 *	15.42 ± 4.82	10.28 ± 0.46	9.78 ± 0.24
Zn	14.43 ± 0.88	13.74 ± 0.79	59.64 ± 6.92 *	32.65 ± 1.53	42.05 ± 1.27	39.76 ± 1.36
Cu	5.98 ± 1.41	6.29 ± 1.16	5.97 ± 1.36	5.05 ± 0.40	9.87 ± 0.53	6.63 ± 1.19
Mn	3.69 ± 0.67	1.61 ± 0.34	4.66 ± 1.51	2.48 ± 0.44	0.63 ± 0.02	0.66 ± 0.01
∑TME	4983.97 ± 319.40	5657.39 ± 1101.56	6943.27 ± 592.93 **	4144.40 ± 33.23	8870.30 ± 763.43	7298.90 ± 167.45
Male						
Na	1626.64 ± 411.30	1567.84 ± 306.79	4430.63 ± 240.01	4502.20 ± 49.07	3459.41 ± 235.32 *	1977.00 ± 317.44
K	2414.57 ± 490.21	2510.66 ± 367.45	3397.72 ± 166.22	2954.14 ± 616.72	3617.96 ± 328.32	2828.30 ± 229.34
Ca	467.16 ± 170.51	780.07 ± 114.32	1129.46 ± 76.44	1064.87 ± 160.05	1084.10 ± 88.74	869.42 ± 150.51
Mg	324.33 ± 79.49	256.74 ± 34.56	554.40 ± 45.71	513.87 ± 5.79	607.20 ± 76.02	456.52 ± 34.59
Fe	108.43 ± 11.12	115.75 ± 17.56	8.47 ± 1.03	8.05 ± 1.40	10.10 ± 1.35	14.08 ± 3.65
Zn	12.20 ± 4.04	14.97 ± 2.14	12.11 ± 1.68	10.38 ± 1.39	31.96 ± 8.21	35.04 ± 0.73
Cu	4.54 ± 1.94	4.04 ± 1.28	5.06 ± 1.03	5.71 ± 0.91	5.90 ± 1.83	5.04 ± 0.69
Mn	3.68 ± 0.61	3.14 ± 0.80	3.55 ± 0.42	3.52 ± 0.44	0.80 ± 0.23	1.21 ± 0.63
∑TME	4961.54 ± 1142.91	5253.21 ± 814.49	9541.40 ± 194.67	9062.75 ± 514.79	8817.43 ± 664.45 *	6186.61 ± 612.24

Notes: Data are presented as means ± standard error (SE) (n = 3). * denotes significant difference (*p* < 0.05). ** denotes extreme significant difference (*p* < 0.01). Abbreviations: IP, *Eriocheir sinensis* reared in intensive pond; SIRW, *Eriocheir sinensis* reared in semi-intensive reed wetland; ∑TME, total mineral elements.

## Data Availability

Data is contained within the article.
